# Epidemiology of Shiga toxin-producing Escherichia coli other than serotype O157:H7 in England, 2016–2023

**DOI:** 10.1099/jmm.0.001947

**Published:** 2025-01-10

**Authors:** Grace King, Claire Jenkins, Iain Hayden, Ella V. Rodwell, Orlagh Quinn, Gauri Godbole, Amy Douglas, Clare Sawyer, Sooria Balasegaram

**Affiliations:** 1 Field Service - South East and London, UK Health Security Agency, London, UK; 2 Gastrointestinal Infections and Food Safety (One Health) Division, UK Health Security Agency, London, UK; 3 Gastrointestinal Bacteria Reference Unit, UK Health Security Agency, London, UK

**Keywords:** clinical symptoms, Shiga toxin-producing *Escherichia coli*, surveillance, virulence profile

## Abstract

**Introduction.** Shiga toxin-producing *Escherichia coli* (STEC) infections are of public health concern as STEC can cause large national foodborne outbreaks of severe gastrointestinal disease, particularly in the young and elderly. In recent years, the implementation of PCR by diagnostic microbiology laboratories has improved the detection of STEC, and there has been an increase in notifications of cases of non-O157 STEC. However, the extent this increase in caseload can be attributed to the improved detection by PCR, or a true increase in non-O157 STEC infections, is unknown.

**Gap Statement.** Epidemiological and microbiological data and analyses describing the trends in non-O157 STEC in England since the implementation of PCR are limited.

**Aim.** Demographic, microbiological and clinical characteristics of non-O157 STEC from 8 years (2016–2023) of laboratory surveillance data were analysed to understand the recent trends in non-O157 serotypes and the incidence of disease in England.

**Methodology.** All human isolates of STEC non-O157 detected between 2016 and 2023 were extracted from the laboratory surveillance system. Microbiological data were analysed and linked to clinical outcomes.

**Results.** There was an almost 10-fold increase in diagnoses of non-O157 STEC from 2016 (*n*=297) to 2023 (*n*=2341). A total of 9378 isolates of non-O157 STEC were detected, comprising 338 different serotypes, and were linked to 9311 individuals. A higher proportion of non-O157 STEC cases were female (56%) and aged between 20 and 39 years (27%). The most common non-O157 serotypes were O26:H11 (16%), O146:H21 (12%), O91:H14 (11%), O128:H2 (6%), O145:H28 (5%) and O103:H2 (4%). STEC O26:H11 was more frequently reported in under 5s than any other age group (38%), whereas the other common serotypes were more frequently isolated from adults. *Stx2a*, which has been associated with greater disease severity, was detected in 18% of cases. Where clinical details were available, 27% of non-O157 cases were admitted to the hospital and 6% developed HUS. Cases of STEC O145:H28 reported a higher rate of hospitalisation than other non-O157 STEC cases. The serotypes most likely to be associated with progression to HUS were O26:H11 (9%) and O145:H28 (7%). STEC harbouring *stx2f* (19%), *stx2a* (11%) and *stx2d* (11%) were most frequently isolated from cases with HUS.

**Conclusion.** The implementation of widespread PCR testing in England has facilitated better surveillance of STEC non-O157, with respect to establishing the true incidence and burden of disease of non-O157 STEC and monitoring the emergence of highly virulent strains.

## Introduction

Shiga-toxin *Escherichia coli* (STEC) is a diverse group of gastrointestinal pathogens defined by the presence of one or more bacteriophage-encoded Shiga toxin genes (*stx*) [1]. All STEC types have the potential to cause gastrointestinal symptoms, such as diarrhoea, abdominal pain and nausea [2]. However, certain types are associated with more severe presentations, including haemorrhagic colitis, vomiting and progression to haemolytic uraemic syndrome (HUS), a potentially fatal systemic condition characterized by acute kidney injury, haemolytic anaemia and thrombocytopenia [3]. There are two types of Shiga toxin (*stx*), *stx1* and *stx2*, and at least ten well-established subtypes (*Stx1a-Stx1d* and *Stx2a-Stx2g*) [4]. STEC that have *stx1a* or *stx2a* are associated with cases reporting bloody diarrhoea and hospitalization, and those that have *stx2a* or *stx2d* have the potential to cause HUS [5]. Outcomes of STEC infections are also linked to the presence and absence of the *eae* gene, with the most severe symptoms being associated with the presence of *eae* in combination with *stx2a* [2].

Previous studies have shown that most STEC types are zoonotic and can cause foodborne outbreaks [6]. Transmission to humans typically occurs through the faecal–oral route by consuming contaminated food or water and through contact with animals and their surroundings [1]. The infectious dose is low (10–100 cells), and person-to-person transmission occurs within households and in institutional settings. Outbreaks of STEC infection have been linked to nursery schools, petting farms and contaminated food products, including unpasteurized dairy products, undercooked meat, salad items and other ready-to-eat produce [7,9].

When STEC emerged in England in the 1980s, the outbreaks of HUS were caused by STEC O157:H7 and, therefore, STEC surveillance strategies, public health actions and interventions focussed on this serotype. STEC O157:H7 can be cultured and identified on selective cefixime tellurite sorbitol MacConkey media, as unlike most other STEC serotypes and other faecal *E. coli*, strains of STEC O157:H7 are resistant to cefixime and tellurite and do not ferment sorbitol. In contrast, molecular methods such as PCR targeting *stx* can be used to detect nearly all STEC serotypes. In 2013, of the approximately 100 diagnostic microbiology laboratories in England, three were using a commercial PCR for the detection of gastrointestinal pathogens including STEC [10]. By 2018, this had increased to 25 laboratories, and recent in-house audits in 2022 and 2023 identified 40 laboratories reporting the PCR results for STEC. Over the last decade, the increase in the number of diagnostic microbiology laboratories implementing the PCR approach has led to an increase in notifications of STEC other than serotype O157:H7 (non-O157 STEC) [10,11].

The epidemiological and microbiological characteristics of non-O157 STEC in England have previously been documented by Vishram et al. in 2021 covering the time frame 2014–2018 [10]. We reviewed the demographic, microbiological and clinical characteristics of non-O157 STEC between 2016 and 2023. The aim of this study was to understand the recent trends in the incidence of non-O157 STEC and how trends have been affected by the increasing use of PCR at the local hospital laboratory level and by the Severe Acute Respiratory Syndrome Corona Virus 2 (SARS-CoV-2) pandemic.

## Methods

### Microbiology

Faecal specimens testing positive for *stx* by PCR, and/or from cases where there is a clinical suspicion of HUS, at local diagnostic laboratories from hospitalized patients, community and asymptomatic cases and contacts of cases of gastrointestinal disease are referred to the Gastrointestinal Bacteria Reference Unit (GBRU), at the UK Health Security Agency (UKHSA) where they are cultured for all STEC serotypes. Since April 2015, all isolates of STEC have been further characterized by whole-genome sequencing, as previously described [12].

### Case definition

A confirmed case of non-O157 STEC was defined as a faecal specimen from a clinical or asymptomatic case, testing (i) PCR positive for *stx* confirmed by GBRU and the serogroup is not O157 or (ii) culture positive for non-O157 STEC. Cases had a sample date between 01 January 2016 and 31 December 2023. In the absence of a sample date, the date of receipt at GBRU was used to determine if the case was in the study time frame.

### Data source

Data from all human cases of non-O157 STEC residents in England and sampled between 01 January 2016 and 31 December 2023 were extracted from the Gastro Data Warehouse (GDW), a UKHSA database that links microbiological data with case demographics. Most cases held two records in GDW, one for PCR results and one for typing result from the culture. These were combined to be one record associated with each sample. Where duplicate results were detected, the earliest positive sample with microbiology typing results was retained.

All registered medical practitioners are legally required to notify local health protection teams (HPT) of both clinically suspected cases of HUS and cases where STEC has been detected. An enhanced surveillance questionnaire (ESQ) for STEC is completed for all relevant cases to obtain a detailed history for the 7 days prior to the onset of illness. The STEC operational guidance aims to ensure that public health follow-up is focused on the cases with the most severe clinical symptoms, and so, in general, cases with mild illness (the absence of bloody diarrhoea, HUS or hospitalization) and those in not high-risk groups were not followed up with an ESQ [13]. The ESQ collects demographic details: risk status; clinical symptoms; exposures including travel, food and water consumption; and environmental exposures. Completed questionnaires are submitted to the national Gastrointestinal Infections and Food Safety (One Health) team at UKHSA to be included in the National Enhanced STEC Surveillance System (NESSS) where the ESQ data for each patient are linked to microbiological typing data. Following the cleaning and deduplication of cases from GDW, the exposure data from NESSS were linked to cases using the laboratory sample unique identifier.

### Data analysis

Variables for analysis included age group, gender, travel history, clinical symptoms and microbiological typing results (serogroup, *stx* subtypes and *eae*). Cases were grouped into the following age categories: 0–4, 5–9, 10–19, 20–39, 40–59, 60–79 and 80 years and over.

Data cleaning and analyses were performed in RStudio version 4.2.2.

## Results

### Overview of case numbers

In England, a total of 9378 isolates of non-O157 STEC were detected from 01 January 2016 to 31 December 2023, linked to 9311 individuals. Of these, 3076/9311 (33.0 %) cases had a PCR-positive faecal specimen; however, STEC could not be isolated with culture methods, and no further microbiology typing was available for these samples. A small proportion of individuals, 67/9311 (0.7 %), had more than one strain of non-O157 STEC detected in their faecal specimen.

With the exception of 2020, and including PCR-positive/culture-negative cases, the total number of cases of non-O157 STEC has increased year on year from 2016 (*n*=297) to 2023 (*n*=2341) (Table S1, available in the online Supplementary Material). Cases of non-O157 STEC increased threefold between 2016 (*n*=297) and 2018 (*n*=938) (Table S1). In 2019, the number of PCR-positive culture-negative cases decreased; however, the number of culture isolations continued to rise, and there was an overall increase in non-O157 STEC cases that year. After the decline in the total number of cases in 2020, notifications increased again, with numbers 2.5 times higher in 2023 (*n*=2341) than in 2018 (*n*=938).

### Overview of the epidemiological data

#### Age and sex

A higher proportion of all non-O157 STEC cases were female (55.9%) than male (44.1%). The age group with the highest proportion of cases was 20–39 years (2527/9363, 27.0%), and there was a greater proportion of non-O157 STEC cases in adults over the age of 20 than those in younger age groups. Information on sex was missing or unknown for 87 cases (0.009%), and date of birth was missing for 15 cases (0.002%).

**Fig. 1.**
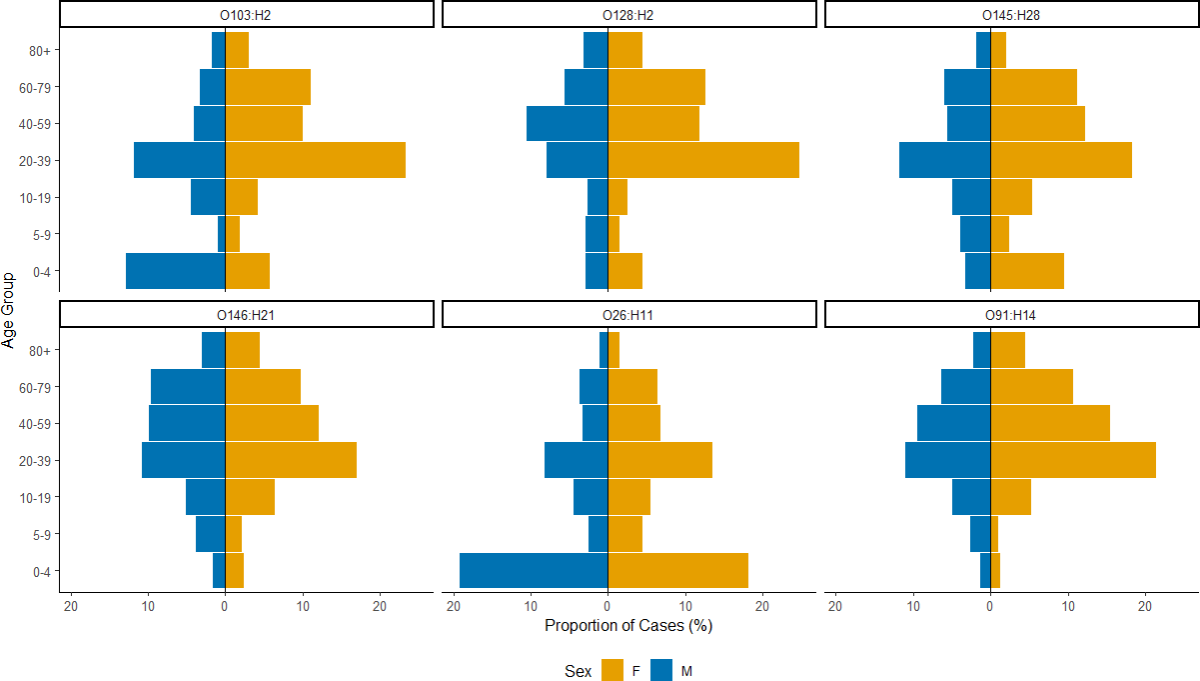
Age–sex breakdown of non-O157 STEC cases in England in the most common serotypes between 01 January 2016 and 31 December 2023.

There was a higher proportion of females compared to males in all six of the most common serotypes, with serotype O128:H2 having the highest proportion of female cases (247/397, 62.2%). This was followed by O145:H28 (180/293, 61.4%), O103:H2 (155/260, 59.6%), O91:H14 (398/658, 60.5%), O26:H11 (577/1021, 56.5%) and, finally, O146:H21 (408/745, 54.8%). STEC O26:H11 was more frequently reported in under 5s than any other age group (385/1023, 37.6%). The age demographics of O26:H11 differ from the age demographics of the other common serotypes, where adult age groups make up the highest proportion of cases. By comparison, in all non-O157 STEC cases, 13.7% of cases made up the under 5 age group. The 20–39 age group had the highest proportion of cases for the rest of the common serotypes; O103:H2 (93/261, 35.6%), O91:H14 (219/660, 33.2%), O128:H2 (132/397, 33.2%), O145:H28 (89/295, 30.2%) and O146:H21 (213/751, 28.4%) (Fig. 1).

### Geographical distribution

Non-O157 STEC cases were observed nationally and in all regions of England over the study period. Postcodes were not available for 3128 cases (3128/9378, 33.4%). Of the 6250 cases where a rural or urban classification could be made based on lower super output area, 1139 cases were resident in a rural setting (1139/6250, 18.2%). A higher proportion of cases residing in rural areas was observed for O26:H11 (165/746, 22.1%) and O145:H28 (52/230, 22.6%), compared to all non-O157 STEC cases. The lowest proportion of cases residing in a rural area were infected with STEC O91:H14 (59/415, 14.2%), followed by O146:H21 (77/484, 15.9%), O128:H2 (41/250, 16.4%) and O103:H2 (35/196, 17.8%).

### International travel

Information on travel outside the UK was available in ESQs for 2932 cases (31.3%). Information was either missing or unknown for the remaining cases administered an ESQ or not recorded for those without an ESQ. International travel in the 7 days before symptom onset was reported in 692 cases of non-O157 STEC (23.6%). Within the most common serotypes, cases of O26:H11 (170/724, 23.4%) and O103:H2 (25/112, 22.3%) reported the most international travel prior to the onset of symptoms. The proportion of international travel reported in the other serotypes was lower: 16.7% for O128:H2, 15.8% for O91:H14, 15.1% for O146:H21 and 13.0% for O145:H28.

### Overview of the microbiological data including serotyping and virulence profiles

Of 6302 cases where STEC was isolated, 6256 cases (99.3%) had serotype information available, and 338 different serotypes were identified. The most common serotypes identified during the study time frame were O26:H11 (1024/6256, 16.4%), O146:H21 (753/6256, 12.0%), O91:H14 (665/6256, 10.6%), O128:H2 (397/6256, 6.4%), O145:H28 (295/6256, 4.7%) and O103:H2 (261/6256, 4.2%).

**Fig. 2.**
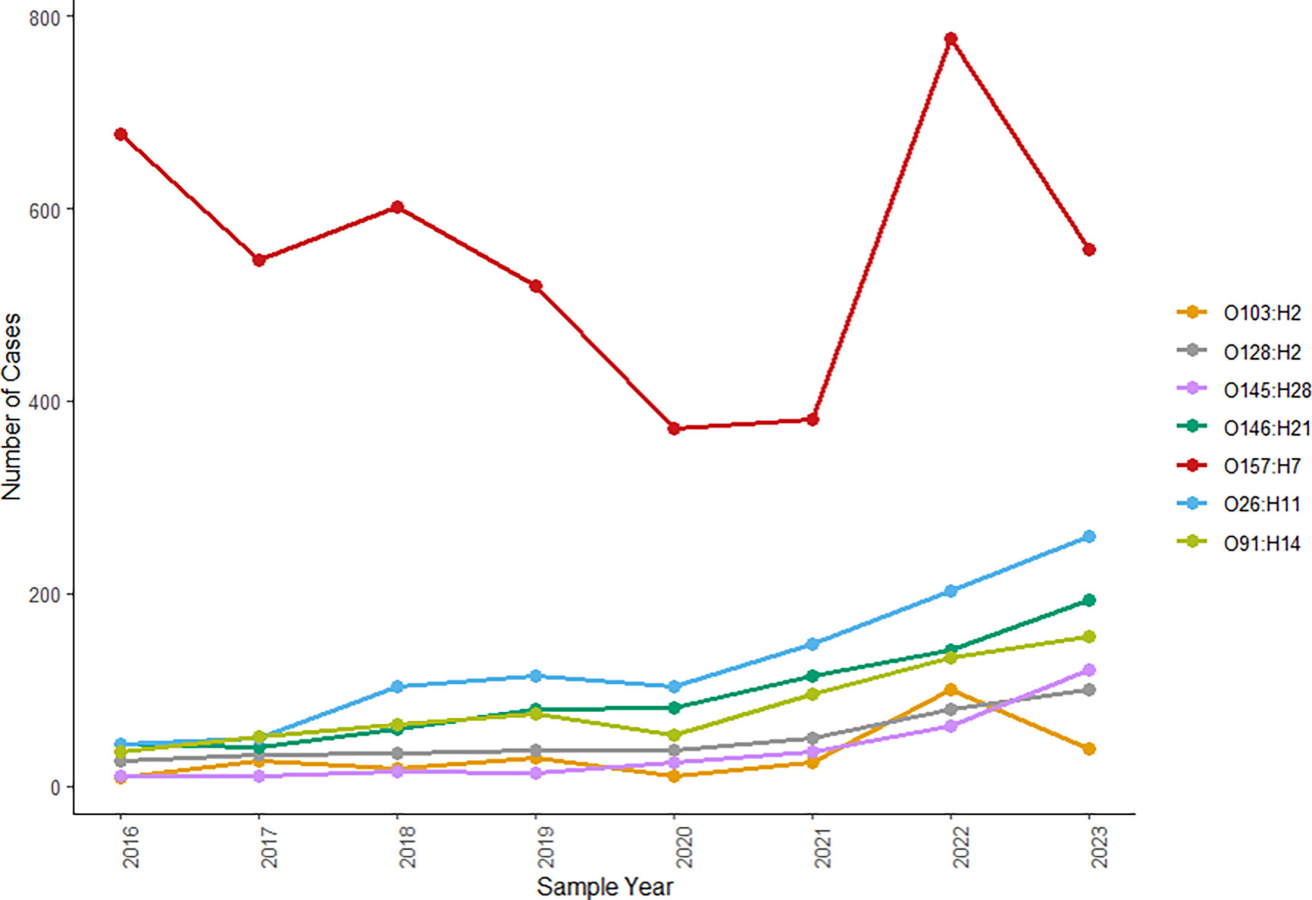
Prevalence of the most common non-O157 STEC serotypes in England, between 01 January 2016 and 31 December 2023, by sample year.

Cases of all six most common serotypes have escalated since 2016, with STEC O145:H28 exhibiting the biggest increase; 2023 saw an 11-fold increase of STEC O145:H28 compared with 2016. STEC O26:H11 saw a fivefold increase in case numbers between 2016 and 2023. Cases numbers of STEC O26:H11, O91:H14 and O103:H2 all declined in 2020, O146:H21 and O128:H2 remained steady, whereas cases of O145:H28 continued to increase (Fig. 2). Data from 2023 revealed a sharp rise in case numbers for all six serotypes described, excluding STEC O103:H2 which decreased in numbers compared to 2022, back to previously observed levels. Notably, STEC O145:H28 cases almost doubled from 2022 to 2023.

**Fig. 3.**
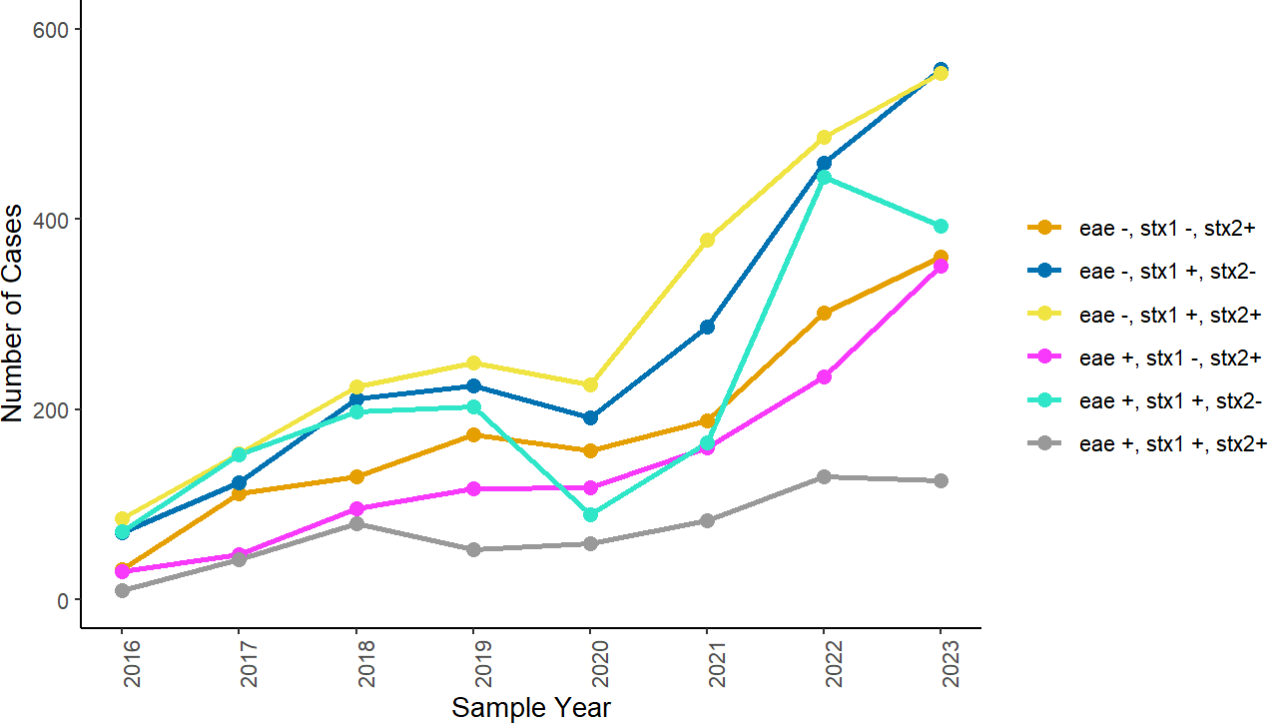
Prevalence of the combinations of *stx1*, *stx2* and *eae* genes in cases of non-O157 STEC in England, between 01 January 2016 and 31 December 2023, by sample year.

*Stx* profiles with or without *eae*, based on the PCR data, were available for all cases of confirmed non-O157 STEC. The highest proportion of isolates had *stx1* (40.9 %, 3840/9378) followed by *stx1*/*stx2* (31.3 %, 2935/9378) and *stx2* (27.8 %, 2603/9378) (Table 1). The *eae* gene was detected in 36.7% (3448/9378) of the cases. Isolates that were *stx1* and *stx2* positive and *eae* negative were the most frequently reported in each year from 2016 to 2022. In 2023, isolates that were only *stx1* positive were the most frequently reported. Between 2021 and 2022, there has been a sharp increase in isolates *eae* positive and *stx1* positive; this combination of genes also saw the steepest decline in 2020 and 2023. The number of isolates that were *eae* positive and *stx2* positive, which has been associated with the severity of disease, has been increasing year on year since 2016 (Fig. 3).

**Table 1.** Prevalence of combinations of *stx1*, *stx2* and *eae* genes in cases of non-O157 STEC in England between 01 January 2016 and 31 December 2023. +

*eae*	*stx1*	*stx2*	*n*	%	HUS (%)
−	+	+	2355	25.11	4 (0.2 %)
−	+	−	2124	22.65	8 (0.4 %)
+	+	−	1716	18.30	6 (0.3 %)
−	−	+	1451	15.47	20 (1.4 %)
+	−	+	1152	12.28	118 (10.2 %)
+	+	+	580	6.18	17 (3.0 %)

+ gene detected; - gene not detected

Of samples with a culture isolated, *stx* profile was available for 6223 (98.7 %) of isolates, and 39 different profiles were detected. *Stx1a* was the most frequently reported *stx* profile (1541/6223, 24.8%), followed by *stx1c* (1067/6223, 17.2%) and *stx1c*/*stx2b* (912/6223, 14.7%) (Table 2). *Stx2a* has been associated with greater disease severity, including the development of HUS. This profile was found in 14 different combinations, totalling 1169 cases (18.9%). The most common *stx* profiles including *stx2a* were *stx2a* only (784/6223, 12.6%) and *stx1a*/*stx2a* (296/6223, 4.8%).

**Table 2.** Prevalence of *stx* profile combinations of non-O157 STEC in England, reported between 01 January 2016 and 31 December 2023

Stx profile combination	*N*	%
*stx1a*	1541	24.76
*stx1c*	1067	17.15
*stx1c*,*stx2b*	912	14.66
*stx2a*	784	12.60
*stx2b*	604	9.71
*stx1a*,*stx2b*	519	8.34
*stx1a*,*stx2a*	296	4.76
*stx2d*	74	1.19
*stx2f*	66	1.06
*stx2e*	57	0.92
*stx2c*	55	0.88
*stx2g*	52	0.84
*stx2a*,*stx2e*	38	0.61
*stx1c*,*stx2b*,*stx2d*	35	0.56
*stx1a*,*stx2c*	17	0.27
*stx2a*,*stx2c*	14	0.22
*stx1a*,*stx2a*,*stx2e*	9	0.14
*stx2b*,*stx2d*	9	0.14
*stx1a*,*stx2a*,*stx2c*	8	0.13
*stx1a*,*stx2d*	7	0.11
*stx1c*,*stx2d*	6	0.10
*stx2c*,*stx2d*	6	0.10
*stx1a*,*stx1c*,*stx2b*	5	0.08
*stx1a*,*stx2b*,*stx2d*	5	0.08
*stx1a*,*stx2a*,*stx2d*	5	0.08
*stx1a*,*stx1c*	4	0.06
*stx2a*,*stx2c*,*stx2d*	4	0.06
*stx2b*,*stx2c*	4	0.06
*stx1c*,*stx2b*,*stx2c*	4	0.06
*stx2a*,*stx2b*	3	0.05
*stx2a*,*stx2d*	3	0.05
*stx1c*,*stx2c*	2	0.03
*stx2a*,*stx2g*	2	0.03
*stx1a*,*stx2a*,*stx2c*,*stx2d*	1	0.02
*stx1a*,*stx2c*,*stx2d*	1	0.02
*stx1a*,*stx2g*	1	0.02
*stx2a*,*stx1c*	1	0.02
*Stx1c*,*stx2a*,*stx2b*	1	0.02
*stx2b*,*stx2c*,*stx2d*	1	0.02

In 2023, whilst absolute numbers remained stable, there was a decrease in the proportion of *stx1a* only reported since 2022 which had increased following the SARS-CoV-2 pandemic. The proportions of other commonly reported profiles remained relatively stable, but a higher proportion of *stx2a* only has been observed in 2023 compared to previous years, accounting for 16.7% of cases. Although a relatively small number of cases have been reported, there appears to be an emergence of *stx2f*, which appears to be increasing since 2020.

### Clinical outcome comparisons for the most common serogroups and virulence profiles

Of the 9378 non-O157 STEC cases, 6271 cases either were not followed up for further public health action or were lost to follow-up (6271/9378, 66.9%), so the proportion of non-O157 STEC cases administered an ESQ was 33.1%. The proportion of cases followed up with completed questionnaires varied by serotype: O145:H28 (259/295, 87.8%), O26:H11 (791/1024, 77.2%), O103:H2 (114/261, 43.7%), O91:H14 (103/222, 29.2%), O146:H21 (216/753, 28.7%) and O128:H2 (110/397, 27.7%). The variation in follow-up is likely attributed to the STEC operational guidance prioritizing public health action for *stx2* and *eae* profile of clinical importance.

The proportion of cases followed up also varied by year. In 2016, the rate of cases followed up with completed questionnaires was the highest (75.8%). The rate of follow-up largely decreased year on year to 2019 (27.8 %) and has gradually decreased again since 2021 (35.9 %) until 2023, when the lowest completion rate was observed (25.3%). The absolute numbers of questionnaires completed were highest in 2022 (663) and 2023 (593), compared to 2016 (225), reflecting the increase in resources required to complete ESQs.

In all non-O157 STEC cases responding to the ESQ, diarrhoea (84.6%) and abdominal pain (67.6%) were the most frequently reported symptoms for all non-O157 STEC cases, and the least reported symptoms for all cases were fever (29.2%) and vomiting (28.5%). The proportion of cases admitted to hospital was 27.0%, 5.6% developed HUS, and there were nine fatal cases (0.3%) (Table 3). The STEC serotypes isolated from the fatal cases were O26:H11 (*n*=3), O54:H45 (*n*=1), O55:H7 (*n*=2), O91:H14 (*n*=1), O145:H28 (*n*=1) and O183:H18 (*n*=1).

**Table 3.** Comparison of clinical outcomes for common non-O157 STEC serotypes

Serotype	Total cases	Total with ESQ (%)	Diarrhoea		Blood in stool		Nausea		Vomiting		Abdominal pain		Fever		Admitted to hospital		HUS*		Fatal	
			*N*	%	*N*	%	*N*	%	*N*	%	*N*	%	*N*	%	*N*	%	*N*	%	*N*	%
Non-O157	9378	3107(33 %)	2630	84.6	1361	43.8	1179	37.9	885	28.5	2100	67.6	908	29.2	840	27.0	173	5.6	9	0.3
O103:H2	261	114(44 %)	105	92.1	72	63.2	48	42.1	28	24.6	82	71.9	27	23.7	29	25.4	0	0.0	0	0.0
O128:H2	397	110(28 %)	86	78.1	35	31.8	43	39.0	11	10.0	76	69.1	35	31.8	15	13.6	1	1.0	0	0.0
O145:H28	295	259(88 %)	234	90.3	177	68.3	130	50.2	94	36.3	216	73.2	86	33.2	122	47.1	17	6.6	1	0.4
O146:H21	753	216(29 %)	170	78.7	62	28.7	75	34.7	50	23.1	139	64.4	57	26.4	28	12.9	1	<0.1	0	0.0
O26:H11	1024	791(77 %)	697	88.1	360	45.5	271	34.3	260	32.9	511	64.6	215	27.2	236	29.8	71	9.0	3	0.4
O91:H14	665	194(29 %)	147	75.8	61	31.4	94	48.5	49	25.2	138	71.1	54	27.8	22	11.3	0	0.0	0	0.0

* Information on HUS was obtained from laboratory forms in addition to the ESQ.

The clinical outcomes and symptoms of non-O157 STEC varied by serotype. Compared to all non-O157 STEC cases reporting symptoms, O145:H28 had the highest proportion of cases admitted to hospital (48.8%) and had high rates of developing HUS (6.6%). Notably, vomiting has been linked to progression to HUS, and O145:H28 observed the highest vomiting rates in this study (36.3%). O26:H11 had the highest proportion of cases developing HUS (9.0%). In addition, O103:H2 had a higher rate of blood in stools reported than all non-O157 STEC (63.2 % vs 43.8 %) and the highest rate of diarrhoea reported (92.1 % vs 84.6 %). Nausea was most reported at the highest rate in O145:H28 (50.2%) and O91:H14 (48.5%). O146:H21 appeared to be less severe than all non-O157 STEC cases for all symptoms (Table 3).

*Stx2f* was the *stx* profile most associated with developing HUS (19.2%), and *stx2a* was the *stx* profile most associated with being hospitalized. Hospitalizations were also higher than that of all non-O157 STEC (27.0%) for *stx2f* (30.8%) and *stx2c* (30.0%). *Stx2a* (11.4%) and *stx2d* (10.7%) also had proportionally higher cases of developing HUS than the proportion for all non-O157 STEC cases (5.6%). None of the case infected with STEC that had *stx1c* or *stx2g *developed HUS. HUS cases that had a *stx* profile including *stx1a* also had *stx2a* in combination for 17 cases (77.3%). Where *eae* was detected alongside *stx2*, the proportion of cases developing HUS was 10.2% (Table 1). All other gene combinations had a proportion of less than 3% (Table 4).

**Table 4.** Comparison of clinical outcomes for *stx* profiles in non-O157 STEC cases

Stx profile	Total cases	Total with ESQ (%)	Number hospitalized	Proportion hospitalized (%)	Number HUS*	Proportion HUS (%)
*stx1a*	2419	1051 (43%)	224	21.3	22	2.1
*stx2b*	2102	652 (31%)	90	13.8	3	0.5
*stx1c*	2037	494 (24%)	75	15.2	0	0.0
*stx2a*	1169	996 (85%)	370	37.1	113	11.4
*stx2d*	157	103 (66%)	26	25.2	11	10.7
*stx2c*	117	80 (68%)	24	30.0	3	3.8
*stx2e*	104	47 (45%)	10	21.3	1	2.1
*stx2f*	66	26 (39%)	8	30.8	5	19.2
*stx2g*	55	19 (35%)	2	10.5	0	0.0

* Information on HUS status was obtained from laboratory forms in addition to the ESQ.

## Discussion

Over the last decade, notifcations of non-O157 STEC have increased substantially in England. The increase in non-O157 STEC cases in England is likely to be partially associated with the implementation of PCR testing at the local laboratory level and changes to the diagnostic algorithms. It is estimated in 2023 that the number of laboratories using this diagnostic algorithm was ~40%. The overall decreasing trends in notifications of cases of STEC O157:H7, despite the improved STEC diagnostic algorithms, suggests that the increasing trend of non-O157 is real, at least in part. If the implementation of the PCR was solely responsible for the increase, there would be an increase observed for all serotypes, including STEC O157:H7. Furthermore, in Wales, PCR testing was implemented in all but one diagnostic laboratory in August 2018, yet we have observed a yearly increase in the number of cases of non-O157 STEC being reported in Wales since that time, despite the number of laboratories implementing the PCR remaining stable (UKHSA and Public Health Wales in-house data). Historically, cattle have been the main animal reservoir of STEC O157:H7 in the UK, however, it is possible that other STEC serotypes may be outcompeting STEC O157:H7 in this niche. UK cattle studies and animal sampling conducted as part of outbreak investigations in England have detected non-O157 STEC in bovine faecal specimens [14,16].

The decrease in non-O157 STEC cases in 2020 are attributed to the introduction and implementations of measures to control the SARS-CoV-2 pandemic. Social distancing measures, closure of open and commercial farms to the public, reduced numbers of children in nursery settings and restrictions on international travel reduced the risks of exposure to STEC. Changes in healthcare-seeking behaviour were also reported during the pandemic with individuals less likely to seek medical help for milder gastrointestinal illnesses [17]. The increase in notifications of non-O157 STEC since 2020 has placed an increased burden on public health and clinical services which is reflected in the proportional decrease in ESQ completion overtime. These increased pressures on services have been particularly notable during multiple large and concurrent outbreaks in 2023 [18]. Cases with mild illness and self-limiting diarrhoea who do not seek medical care will not be tested, and the true burden of non-O157 STEC is likely to be much higher than the caseload highlighted in this study.

STEC O26:H11 has emerged to be the most frequently reported serotype of non-O157 STEC since 2019, followed by O146:H21, O91:H14, O128:H2, O145:H28 and O103:H2. The number of cases in all six serotypes listed increased year on year since 2020, with the exception of O103:H2 where a decrease was observed between 2022 and 2023. Excluding the 259 cases associated with a large national outbreak of O157 STEC in 2022, unlike non-O157 STEC, there has been an overall decline in cases of STEC O157:H7 since 2016. STEC O26:H11 has been associated with nursery outbreaks, which may explain the higher proportion of cases in children compared to the other non-O157 serotypes [19]. Between 2014 and 2018, STEC O117:H7 was among the most common non-O157 STEC serotypes causing human infection; however, this study has highlighted the emergence of STEC O145:H28 as one of the most common serotypes, overtaking STEC O117:H7 [3]. STEC O145:H28 had the lowest association with international travel in this study and recently has been associated with national foodborne outbreaks linked to unpasteurized cheese, the salad component of pre-packed sandwiches and other ready-to-eat food items [20,21]. The increase in O145:H28 in 2023 has been largely attributed to three nationally investigated outbreaks, with around 70% of cases being part of an outbreak.

Non-O157 STEC has been more frequently detected in females than males, and this distribution of sex has also been observed in the most frequently reported serotypes. This has been attributed to the increased risk of exposure to STEC during food preparation and childcare, and females being more likely to consume ready-to-eat foods often contaminated with STEC such as fresh produce and more likely to engage in healthcare-seeking behaviours.

In our study, infection with STEC O26:H11 or STEC O145:H28 was associated with a greater risk of severe disease, evidenced by higher rates of hospitalization and development of HUS. STEC O26:H11 was most commonly observed in children, who are at greater risk of developing HUS. Argentina has reported the highest rates of HUS infections globally, and in 2018/2019, an increase in the percentage of HUS caused by STEC O145 was observed [22]. Our study also provides further evidence that *stx2a* is associated with high-risk outcomes (hospitalizations and HUS), and similar findings were also observed for *stx2d* and association with HUS. Although *stx2c* was less frequently associated with HUS than *stx2a*, it presented a similarly high proportion of hospitalizations. These *stx* profiles still present as higher-risk strains, and these findings provide further evidence that the operational guidance for the management of STEC correctly prioritizes resources to manage strains linked to severe outcomes and the importance that these profiles continue to be followed up by HPTs.

Our study provides increasing evidence that *stx2f* is an emerging high-risk *stx* subtype, despite being the cause of a small proportion of non-O157 STEC cases [23]. Compared to other *stx* subtypes, *stx2f* had the highest proportion of cases developing HUS. We recommend that *stx2f* be included as a high-risk strain and followed up with public health action. The findings of our study show the importance of whole genome sequencing for predicting the virulence profile of the strains including emerging *stx* subtypes like *stx2f*; it demonstrates that virulence factors are the key to predicting the clinical outcomes and should form the basis of clinical and public health risk assessments.

This study highlighted different levels of severity of clinical outcomes for different serotypes and *stx* profiles. The UKHSA operational guidance for following up cases of STEC prioritizes the higher risk serotypes (such as O26:H11) and *stx* subtypes (*stx2a*, *stx2c* and *stx2d*). As a result, details on clinical outcomes were only available for 33.1% of cases, as the remaining cases did not fulfil the required follow-up criteria. The decrease in the proportional ESQ completion over time may also be partially attributed to the increasing volume of cases placing increase have more severe infections. Clinical details from patients with serotypes causing milder infections were not captured in this study. Furthermore, details of symptoms and hospitalizations were only captured at the time the questionnaire is administered. Additional symptoms and subsequent hospitalizations that occurred after the ESQ was completed, were not be captured in this study. This is particularly concerning when capturing outcome information on HUS, as there is a median delay of 6 days from GI symptoms to hospitalization or HUS diagnosis [24].

Over the last decade, notifications of cases infected with non-O157 STEC in England have increased. We concluded that this trend is due partly to a more widespread use of the PCR approach at the local hospital level in England, but also represents a true increase in the clinical and public health burden of non-O157 STEC. Prior to the implementation of the PCR assays and the subsequent improvements to STEC surveillance, the burden of infectious intestinal disease captured here occurred under the surveillance radar. This study highlights the importance of investing in and maintaining resolution diagnostic tools such as WGS, which are able to reliably predict virulence profiles and enable early signalling of the most pathogenic strains which are likely to cause severe illness and outbreaks. Ongoing high-resolution genomic surveillance of all STEC serotypes will enable us to identify emerging zoonotic gastrointestinal pathogens and to proactively implement interventions to protect public health.

## supplementary material

10.1099/jmm.0.001947 Uncited Table S1.

## Abbreviations

ESQ: enhanced surveillance questionnaire
GBRU: Gastrointestinal Bacteria Reference Unit
GDW: Gastro Data Warehouse
HPT: health protection team
HUS: haemolytic uraemic syndrome
NESSS: National Enhanced STEC Surveillance System
SARS-CoV-2: Severe Acute Respiratory Syndrome Corona Virus 2
STEC: Shiga toxin-producing *Escherichia coli*
UKHSA: UK Health Security Agency
WGS: whole genome sequencing
